# Aging Airways: between Normal and Disease. A Multidimensional Diagnostic Approach by Combining Clinical, Functional, and Imaging Data

**DOI:** 10.14336/AD.2016.1215

**Published:** 2017-07-21

**Authors:** Mariaelena Occhipinti, Anna Rita Larici, Lorenzo Bonomo, Raffaele Antonelli Incalzi

**Affiliations:** ^1^Department of Experimental and Clinical Medicine, Careggi Hospital, University of Florence, 50134 Florence, Italy; ^2^Department of Radiological Sciences, Gemelli Hospital, Catholic University of Sacred Heart, 00168 Roma, Italy; ^3^Department of Geriatrics, Campus Bio Medico University, 00128 Roma, Italy

**Keywords:** aging airways, spirometry, multidimensional approach, post-processing imaging techniques, parametric response maps

## Abstract

The lack of data on lung function decline in the aging process as well as the lack of gold standards to define obstructive and restrictive respiratory disease in older people point out the need for a multidimensional assessment and interpretation of the aging airways. By integrating clinical data together with morphologic and morphometric findings clinicians can assess the airways with a more comprehensive perspective, helpful in the interpretation of the “grey zone” between normal aging and disease. This review focuses on the value of a multidimensional approach in the study of the aging airways, including clinical findings, respiratory function tests, and imaging as parts of a whole. Nowadays this multidimensional diagnostic approach can be used in daily clinical practice. In next future, it can be implemented by the analysis of exhaled gases, post-processing imaging techniques, and genetic analysis, that will hopefully reduce the gaps in knowledge of normal aging and airway disease in older people.

The aging process affects both structure and function of airways, lung parenchyma, and respiratory muscles. This process results in changes of the respiratory flows, with dynamic lung volumes decreasing and residual volume increasing progressively with age. Normal standards must be tailored to age accordingly. Indeed, the recent European Respiratory Society/American Thoracic Society Task Force for COPD Research Statement highlighted the importance of “aging” in defining airway disease by requesting new studies that “evaluate the impact of age on the importance of identifying an airflow limitation” [1]. Clinical evaluation together with pulmonary function tests (PFTs) is necessary to characterize the pattern of airway disease as well as its severity. However, spirometry is underused and difficult to perform in older people and there is no spirometric gold standard specific in this population for the diagnosis of obstructive disease. Imaging can to some extent integrate or also substitute for respiratory function data in highly problematic cases, providing important clinical information.

The limitations of PFTs and imaging should promote efforts to combine both into a multidimensional assessment of the older respiratory patient, by integrating clinical data together with morphologic and morphometric findings. Nowadays such a multidimensional approach can be used in our daily clinical practice and in next future it can be implemented by new diagnostic non-invasive methods, such as analysis of exhaled gases, post-processing imaging techniques, and genetic analysis.

## The aging airways

The aging process of the airways manifests with many features, ranging from a decrease in dynamic lung volumes and mucociliary clearance to an increase in bronchial responsiveness.

### Volumes

Aging is characterized by a gradual decrease in the surface area of airspace wall per unit of lung volume and enlargement of airspaces [2]. This finding was originally defined as “senile emphysema”, but it actually differs from the pathological condition of emphysema. Indeed, in the normal aging lung alveolar septa are intact and the elastic network supporting the small airways is only partially damaged, with alveolar sacs modestly enlarged [3, 4]. Accordingly, the age-related increase in the residual volume to total lung capacity ratio does not reflect a true emphysematous change of the aging lung. Indeed, the fact that arterial oxygen tension and alveolar-arterial oxygen tension difference are stable in the healthy older people testifies that gas exchanges are well preserved even in the very old [5, 6]. Thus, the age-related decline in lung volumes does not affect the ventilation-to-perfusion ratio [7].

Individual genetic factors as well as modifiable factors, including smoking and environmental pollution, variably affect the decline of lung volume with age and the related shortening of telomeres in bronchial, alveolar and endothelial pulmonary cells [8]. The features of an aging lung may indeed be present even in adult or young-adult people with an unfavorable risk profile. Among modifiable factors, smoking plays an important role, with the yearly loss of dynamic lung volumes increased up to 100% in heavy smokers, from the usual 20-30 ml up to 60 ml [3].

At any age, the measured lung volume may be considered to depend upon the achieved volumes at the time of lung maturity, i.e. at age 20-25 years, and the rate of decline. Thus, growing optimally and adhering to a healthy life style in the adulthood are expected to guarantee for the best respiratory function even in the very old.

The heterogeneity in lung function decline increases in the diseased lungs. In the “Eclipse” study a dramatic inter-individual variability in the yearly decline of forced expiratory volume in 1 second (FEV_1_) has been observed in patients with chronic obstructive pulmonary disease (COPD) (mean age 63±7, range 40-75 years) [9]. The median yearly loss of FEV_1_ was 32 ml, with 20% of patients loosing over 60 ml per year and 18% of patients with no change or only a small positive change in FEV1 over a 3-years period [9].

### Mucociliary clearance

Aging is characterized by weakened pulmonary immune defenses and dramatically decreased mucociliary clearance [3]. These changes result in an increased susceptibility to lung infections as well as in an increase in incidentally found imaging findings, such as bronchiectases, cysts, scars, and bronchial dilation. These findings are variably present in older patients without a clear history of respiratory disease.

### Bronchial responsiveness

Aging is associated with increased bronchial responsiveness to cholinergic drugs or non-pharmacologic stimuli. However, this does not translate into an age-related increase in the prevalence of asthma. Asthma is more common in young adults, and after the age of 60 years it is phenotypically different: uncommon history of allergic disease, nocturnal and early morning symptoms, poor immediate response to the bronchodilator. Thus, asthma is frequently unrecognized in this population or misclassified as COPD [10, 11]. Bronchial responsiveness is also associated with accelerated lung volume decline and correlates inversely with airway caliber [12].

### Other changes

Although not directly related to the airways, the age-related decline in respiratory muscle strength (about 1% each year after age 30 [13]) accounts for decreased ventilatory reserve, limiting the response to the exercise and to stressing conditions, such as pneumonia or ARDS. Furthermore, pulmonary vascular reserve declines with age [14] and even subjects with well-preserved respiratory function are at risk of respiratory muscle exhaustion at rest and of acute respiratory failure in stressing conditions.

## Spirometry: to the limits and beyond

Clinical evaluation together with PFTs are necessary to characterize the pattern of airway disease as well as its severity. Age-related decrease in dynamic lung volumes can be assessed through the predicted values of FEV_1_ and forced vital capacity (FVC), that present less inter-individual variability than mid-expiratory and end-expiratory flows [15]. The classic fixed *FEV_1_/FVC ratio* is a very simple definition of bronchial obstruction, but it physiologically declines with age [2] and can lead to an over-diagnosis of bronchial obstruction in older people [16]. Indeed, in the Sa.R.A. (Salute Respiratoria nell’Anziano - Respiratory health in older people) study, involving people aged 65 years and over, the fifth percentile of the FEV1/FVC distribution was 0.65 for men and 0.67 for women. Taking into account the classic fixed FEV_1_/FVC ratio of 0.70 as limit for obstruction, the 15% of the population over 65 would have been inappropriately classified as obstructed [17]. Thus, the Lower Limit of Normal (LLN) has been proposed as an alternative to the fixed FEV_1_/FVC ratio, independent from clinical factors. However, there are still controversies, as demonstrated by two different studies in populations older than 65 years of age. Mannino et al. [18] found that the fixed FEV_1_/FVC ratio has greater prognostic implications than the LLN, whereas Luoto et al. [19] achieved opposite results. Although the analysis of reasons accounting for this discrepancy is beyond the scope of this review, differences in smoking habits, ethnicity, and years of follow-up between the populations studied should be taken into account.

**Figure 1.**
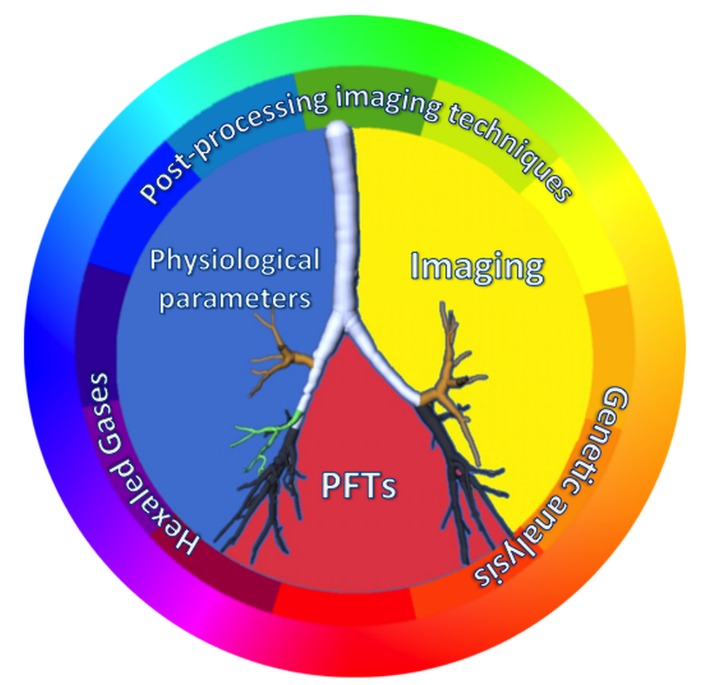
Multidimensional approach Likewise, the match of primary colours generates the entire colour wheel, the match of physiological parameters, imaging, and pulmonary function tests (PFTs) allows studying the multiple facets of the airways in older people. They could be studied even deeply in next future by adding to this multidimensional approach the data provided by analysis of exhaled gases, post-processing imaging techniques, and genetic analysis.

Another score that could be used in older people is the *Z score*, a standardized score of FEV_1_ independent from age, gender, and height. Applied to spirometric indexes, Z score looks like a promising diagnostic and classificatory tool in geriatric population [18]. Indeed, the Z score correlates with important clinical outcomes, such as respiratory symptoms in general, dyspnea-on-exertion, and mortality [20]. However, the available data on literature do not allow drawing definitive conclusions about the spirometric gold standard for the diagnosis of COPD in older people. Moreover, spirometry is underused in older people, as it could be difficult to perform especially by disabled people. In the Sa.R.A. study one out of five people over the age of 64 years could not meet American Thoracic Society/European Respiratory Society acceptability and/or reproducibility criteria for spirometry [21]. Lower education, cognitive impairment, and polypharmacy were independent predictors among the not achievers. Measuring forced expiratory volume in 6 seconds (*FEV_6_*) could expand the population with a diagnostic spirometry, but still 18% of probands have a bad quality test [22]. These issues about spirometry affect the data on longitudinal changes in FEV_1_ in COPD populations: all the available information stems from pharmacological trials on highly selected individuals. Thus, there is a lack of reliable information on lung function decline in frail and dependent people. This points out the need for a multidimensional assessment and interpretation of the aging airways by integrating spirometric data together with morphologic and morphometric findings and, in next future, with data provided by innovative diagnostic methods (see section “New horizons”). The general systems theory of von Bertalanffy [23] inspires for a multidimensional assessment based on a comparative analysis of PFTs, imaging, and physiological parameters as a function of age (Fig. 1).

*Imaging* can integrate the functional assessment in selected conditions and can partly compensate for the vast proportion of older people unable to perform high quality PFTs, being less dependent from patient’s cooperation than PFTs and suitable to many probands. Although chest X-ray (CXR) is the first step in the evaluation of airway disease, findings are often subtle and can be missed. Therefore, computed tomography (CT) is the main imaging technique to assess the airways. Morphometric analysis of the small airways lacks a well-defined functional equivalent and this is especially true in older people. Nevertheless, imaging provides a structured morphometric analysis of the aged lung and useful information on large airways, which lack a respiratory function equivalent.

Although imaging cannot directly inform about the respiratory function, indirect insight is valuable. For instance, imaging findings of air-trapping at expiratory scans are clearly consistent with small airway disease, that can be completely silent at PFTs. Furthermore, a negative CT scan can be very helpful in excluding a pulmonary origin of respiratory symptoms, suggesting alternative diagnoses. Finally, imaging can provide additional information about diaphragm mass and mobility, pericardial effusion, rib and vertebral fractures, ascites, and nervous compression, that all influence respiratory function.

**Figure 2.**
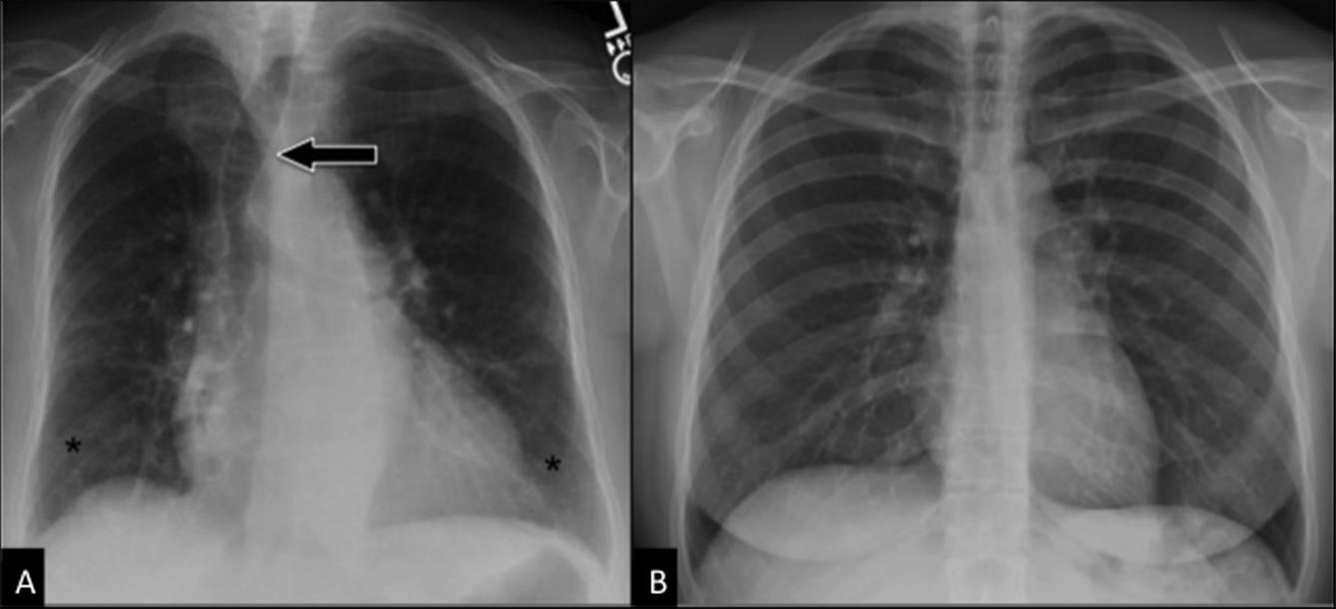
Aging airways Comparison between “normal” CXR in an elderly subject (**A**) and in a young adult (**B**). Postero-anterior CXR in an 87 years-old woman (**A**) shows displacement of the trachea *(arrow)* to the right side, calcifications of the tracheobronchial cartilage, symmetrical bilateral reduction in lung vascularity (more prominent in middle-upper regions), linear and reticular opacities in lung bases *(asterisks)*, and bronchial wall thickening. All these findings cannot be seen in the younger adult (**B**, 45 years-old woman).

## Imaging findings between normal aging and clinically relevant airway disease

In older people, a grey zone exists between normal aging and disease, generating diagnostic issues to both clinicians and radiologists. The definition of normal radiological appearance of the airways in older people would be helpful in daily practice as well as in research. However, literature data on radiological appearance of aging airways in asymptomatic older individuals is scarce and dispersed [24, 25]. Specifics for accurate imaging evaluation in older people are summarized in Table 1.

Common findings in imaging examinations of *aging airways* without a clinical relevance include the followings (Fig. 2):

- *Calcifications of tracheobronchial cartilage*, evident at CXR and MDCT scans. They occur in up to 65% of men and 41% of women in the age range of 60-79 years [26].
- *Displacement of the trachea to the right-side* usual in more than 30% of cases, due to the enlargement of the atherosclerotic aortic arch [27].
- *Tracheal dilation and higher collapsibility.* In a recent study O’Donnell, et al. [28] analyzed tracheal dimensions of 81 subjects from 25 to 75 years at both end-inspiration and during forced expiration on CT scan. Relative to younger ones, older men had an average 11% greater cross-sectional area at end-inspiration and 40% smaller cross-sectional area during forced expiration. Conversely, older women had 14% greater mean tracheal area at end-inspiration, but no difference in area at expiration. Data at end-inspiration confirm the results of previously published studies on tracheal dimensions in CXR [29-31]. In particular, Breatnacht et al. [29] studying a group of patients from 10 to 79 years of age revealed changes in tracheal diameters till the forth decade of life and in the cross-sectional tracheal shape from round to ovoid. From the third and through the eighth decade, the coronal and sagittal diameters were statistically greater in men than in women: in the seventh decade of life the mean sagittal and coronal diameters were 20.7 and 19.5mm in men and 17.2 and 16.8mm in women respectively, and in the eight decades of life the mean sagittal and coronal diameters were 20.8 and 19.7mm in men and 16.5 and 16.4mm in women, respectively.
- *Bronchial dilation.* In 1976 a physiological study by Gibson et al. reported an age-related increase in unstressed airway dimensions and a loss of elastic recoil of both airways and lung parenchyma, qualitatively similar to that found in other tissues [32]. More recently, a radiological study by Matsuoka et al. [33] observed a mean value of bronchoarterial ratio in subjects older than 65 years significantly higher than the one obtained in younger subjects (0.78 versus 0.61-0.70). Moreover, in 41% of cases a normal bronchoarterial ratio overlaps with the ratio considered to be the threshold for bronchiectases [33]. The authors postulated the role of hypoxic bronchodilatation and vasoconstriction as the reason for the increase in the bronchoarterial ratio with aging. However, hypoxia is not a physiologically age-related event and other age-dependent alterations in bronchial structure with weakening in elastic recoil would account for bronchial shrinkage rather than dilation. Therefore, the interpretation of this finding remains unclear.
- *Bronchial wall thickening*. Vikgren et al. [34] studied 92 healthy subjects 61-62 years old and followed them up to 6 years later. They found bronchial wall thickening in 35% of never-smoker subjects at baseline with a substantial increase to 69% after 6 years’ follow-up, due to the aging process.
- *Air-trapping.* The frequency of air trapping significantly increases with age, being present in 76% of subjects at the age of 61 or older [35]. Moreover, the extent of air-trapping has a significant correlation with age as well as with a smoking history of more than 10 pack-years [35]. By using a new technique of co-registration analysis of paired inspiratory and expiratory CT scans (see section “New Horizons”) an association between increasing age and more functional small airway abnormality (PRM^FSA^) was found, with an increase in PRM^FSA^ per decade of age, ranging from 3.6% in the 5^th^ decade to 12.7% in the 8^th^ decade [36]. Moreover, the contribution of aging to greater PRM^FSA^ was different in presence of established obstruction, with a 4.2% higher PRM^FSA^ per decade among subjects with obstruction, versus a 2.7% higher PRM^FSA^ per decade among those without airflow obstruction [36].
- *Secretions.* They are a common finding in elderly airways, usually presenting low attenuation and “bubbly” appearance, due to commixture with air. They more commonly occur along the dependent portion of the airway.
- *Senile lung.* Aging modifications of the lower airways superimposed on an increase of pulmonary supporting interstitial connective tissue and on a homogeneous reduction of pulmonary vascularization create the so-called “dirty chest” on CXR [27]. This is characterized by a reduction in lung opacity and vascularity, linear and reticular opacities, cystic air spaces, bronchial thickening, and bronchial dilation [37]. These findings are very common in the elderly and do not represent frank disease.

**Figure 3.**
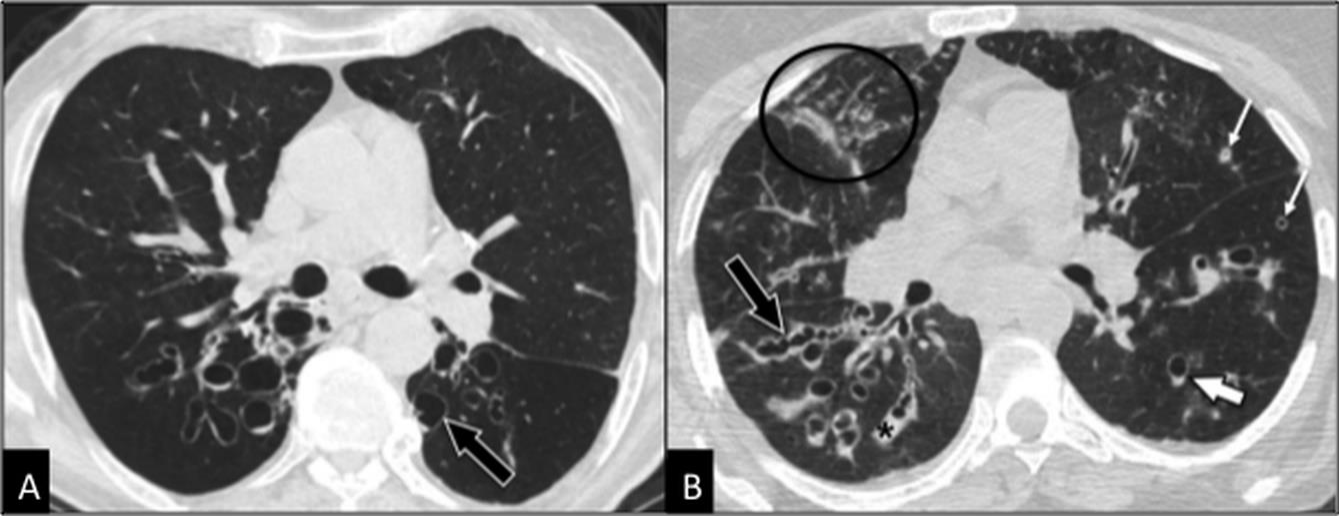
Clinically relevant airway disease Comparison between bronchiectases (**A**) and bronchiolitis due to underlying bronchiectases (b). CT is diagnostic in both cases showing in the first case (**A**) dilated bronchi with cystic appearance *(arrow)* whereas in the second case (**B**) dilated bronchi with cylindrical and varicose *(black arrow)* appearance, together with bronchial mucoid impaction *(black asterisk)*, thickening of bronchial walls *(thin white arrows)*, centrilobular nodules and consolidations *(circle)*. The patchy and asymmetric distribution of the above-mentioned findings is typical of infectious bronchiolitis. Note the “signet ring sign” *(thick white arrow)* typical of bronchiectasis, due to the greater diameter of the bronchus in comparison to the adjacent artery.

*Clinically relevant airway diseases* occurring more commonly in older people than in younger adults are *COPD, CPFE, bronchiectases (Fig. 3), bronchiolitis (Fig. 3B), expiratory central airway collapse, bronchial anthracofibrosis, broncholithiasis*, and *neoplasms*. The main features and the clinical relevance of these diseases in older people are summarized in Table 2. There are also airway diseases occurring less commonly than in adults, such as infiltrative diseases like sarcoidosis.

On one hand, physicians prescribe an imaging study of the airways when a clinical suspicion of disease exists to characterize the disease itself. On the other hand, the identification of imaging findings of airway disease cannot directly translate into prescription of respiratory drugs. For example, in case of CT-defined COPD there is no evidence that the cost/benefit ratio of daily COPD inhalers is favorable for these patients unless their FEV_1_ is below 60% predicted [38].

**Figure 4.**
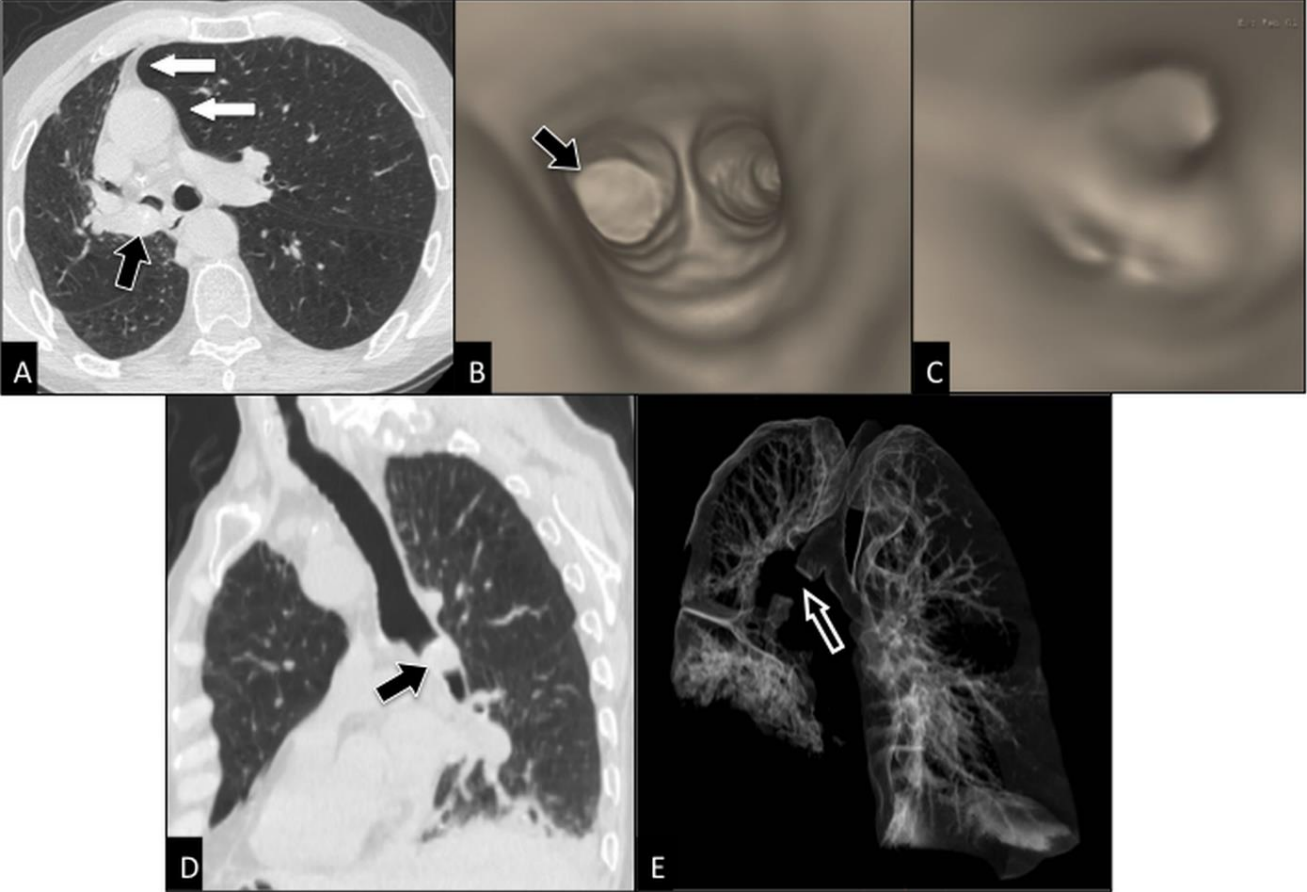
Post-processing imaging techniques: 2D and 3D reconstruction images Axial CT scan (**A**) shows an endobronchial mass *(black arrow)* within right main bronchus in a 78 years-old man with squamous cell carcinoma, causing partial collapse of right lung with displacement of mediastinum towards right *(white arrows)*. 3D virtual bronchoscopy (**B, C**) reproduces the bronchoscopic appearance of the mass occluding right main bronchus *(arrow in b)*, but it allows more than bronchoscopy the visualization of the patent bronchi distal to obstruction site (**C**). Curved 2D MPR (multiplanar reconstructions) image (**D**) better depicts the cranio-caudal extent of the mass *(arrow)* into right main bronchus than the axial image (**A**). CT bronchography (**E**) allows a global visualization of the aerated lung and shows the site of interruption *(arrow)* of the tracheobronchial tree.

## An integrated diagnostic approach: our daily horizon in clinical practice

Older patients with dyspnea, cough, wheezing, or recurrent respiratory infections should undergo an accurate clinical evaluation, including *PFTs*. In many cases this approach allows the diagnosis of well-defined respiratory conditions with an obstructive pattern, more commonly in patients with COPD and asthma, and a restrictive pattern, more commonly in patients with interstitial lung disease and rib cage deformity. However, conditions with univocally categorized obstructive or restrictive pattern on PFTs may indeed be atypical in older patients. On the one hand, bronchial obstruction may be only to some extent reversible and clinical presentation in older asthmatics may be misleading (see above) [10]. On the other hand, older people can present mixed patterns on PFTs, i.e. to a variable extent obstructive and restrictive, more commonly than younger adults. For instance, an older patient with COPD, congestive heart failure, and sarcopenia is expected to have a mixed pattern, but it is difficult to dissect out the individual contribution of each separate condition on a purely clinical basis. *Imaging* may help in identifying and staging comorbid conditions contributing to such a mixed pattern. However, more commonly it is the combination of clinical data and imaging findings that permits a correct diagnosis and management. Overall, patients with a restrictive pattern at PFTs need to undergo a chest CT scan to diagnose any interstitial lung disease or extra-pulmonary disorders, such as chest wall abnormalities, rib and vertebral fractures, pleural effusion and pleural disease, diaphragmatic dyskinesia, and sarcopenia (obesity and chronic heart failure can also cause a restrictive pattern, but clinical presentation is diagnostic). Conversely, patients presenting with an obstructive pattern at PFTs need a CXR for a first diagnostic evaluation, followed by a chest CT scan in all those patients requiring a deep insight. Indeed, patients with large airway disease require a chest CT scan to assess the presence of stenoses, broncholithiasis, relapsing polychondritis, trachea-bronchomalacia, and excessive dynamic airway collapse (EDAC). In particular, patients with suspected large airway disease and without imaging findings of large airway disease at CT performed at end-inspiration need a specific expiratory scan to diagnose tracheobronchomalacia, EDAC, or relapsing polychondritis. Patients with an obstructive pattern at PFTs with lower airway disease need to be studied by CT scan, as it can reveal bronchiolitis, bronchiectasis, bronchial thickening in cardiac failure, as well as COPD with its specific phenotype. Patients with asthma represent an exception, as they can be studied clinically and on CXR. Variations should be considered according to individual conditions and prognosis as well as timing and circumstances of CT examination should be carefully scrutinized. Findings of airway disease incidentally found on CXR or CT performed for other reasons require afterwards an accurate clinical evaluation. This can occur in case of bronchiectases, diverticula, broncholithiasis, and bronchiolitis. For instance, bronchiectases may be completely silent, mainly if located in the apical regions, or may cause recurrent or chronic respiratory symptoms generically ascribed to bronchitis before performing CT scan.

Diffusing lung capacity of carbon monoxide (DLco) can help in daily practice as reflects gas exchanges through the alveolar-capillary membrane and can be used in assessing both restrictive and obstructive airway disease. DLco is an important determinant of the clinical phenotype of COPD, being more reduced in emphysema-predominant phenotype than in airway-predominant disease [39].

**Table 1:** Imaging specifics for chest CT evaluation of airway disease in older people.

MDCT	CT scans to better assess the airways (from the trachea to the most distal visible bronchi) are multidetector CT scans (MDCT), widely spread in Western countries. MDCT scans allow generating 2D multiplanar and 3D reconstructions of the airways.
Patient positioning	Can be challenging in those with musculoskeletal problems. Rotation of the gantry can be helpful in obtaining axial scans even in severe dorsal kyphosis.
Patient Dose	Moderate radiation doses are acceptable at inspiratory CT scan (<10 mSv). Expiratory CT may be performed with lower radiation exposure (tube current ≤ 50 mAs) [43].
Motion artifacts	MDCT takes only a few seconds to scan the entire chest, reducing or avoiding motion artifacts even in breathless patients.
Intravenous contrast media	Routinely not recommended. Required in known or suspected airway neoplasms, paratracheal masses causing airway obstruction, and in suspected pulmonary embolism. In case of contrast administration, glomerular filtration rate should always be performed, as normal values of creatinine in the blood for the adult population are not consistent with a good renal function in older population.
Breathing instructions	Fundamental to avoid motion artifacts and misleading diagnoses of airway disease. Issues in accurately following breathing instructions are more common than in younger adults, because of ear loss, dyspnea, cough, weakness of chest wall muscles, or cognitive impairment. A better compliance in following breathing instructions can be obtained by showing the procedure to the patient as well as by careful coaching the patient before and during the examination. Coaching consists of making the old patient comfortable with the instructions, ensuring him that the instructions will be repeated during the examination and he does not have to remember everything, and giving him the chance to try the protocol more times as needed [55].

(MDCT= Multidetector Computed Tomography; 2D= two dimensional; 3D= three dimensional; mSv= millisievert; mAs= milliamper/second)

Bronchoscopy can provide additional information to clinical and imaging data in the study of the airways also in elderly subjects, as it can be performed safely and effectively in this subset of population, with only one report of a greater incidence of adverse reactions in patients 80 years or older [40]. Bronchoscopy allows also the study of wall changes and endobronchial mucosal vasculature in COPD patients [41]. Bronchoscopy-related procedures, such as endobronchial ultrasounds and transbronchial needle aspiration, are also safe and effective, even though they are invasive and might not be well tolerated by the elderly [42]. Endobronchial interventions including radiotherapy, laser therapy, photodynamic therapy, cryotherapy, and airway stenting can be performed in cases of large airway stenoses as well as neoplasms, with either palliative or therapeutic aim [43]. They can also represent a “bridge” treatment of severe dyspnea and hemoptysis before surgery. Finally, in COPD patients lung volume reduction performed via bronchoscopy can produce a significant improvement in lung function in patients with heterogeneous emphysema and intact interlobar fissures by using endobronchial valves [44]. Coils, vapor thermal ablation, and sclerosant agents are alternative to endobronchial valves, but they should be used in more specific settings [45]. Chest CT scan with 2D and 3D reconstructions is fundamental in all types of bronchoscopy-related procedures, in both pre-treatment evaluation and follow-up.

**Figure 5.**
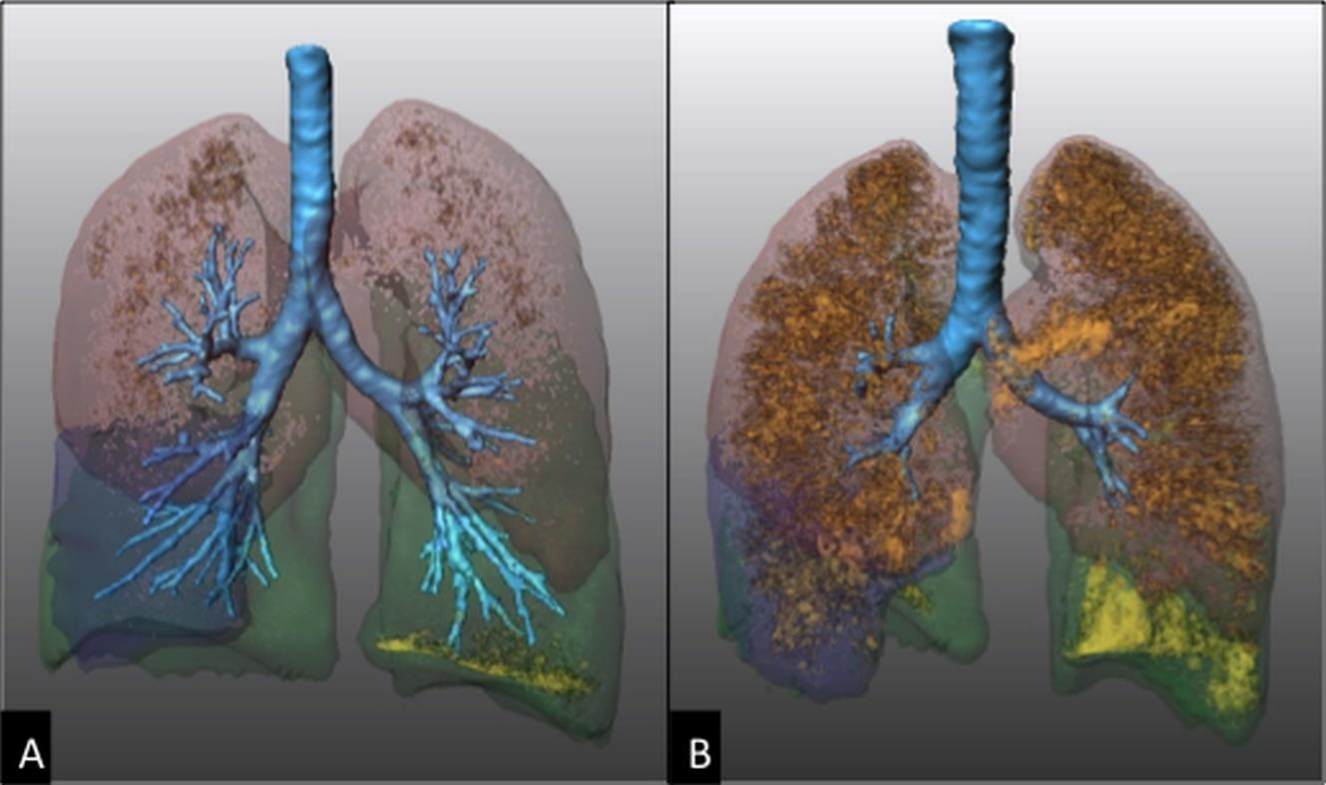
Post-processing imaging techniques: quantification of emphysema and air-trapping Quantification in a 73 years-old woman with COPD by using dedicated software of semi-automatic lung volume segmentation (Franhofer MeVis, Germany, DE). Volume rendering CT images at end-inspiration (**A**) and end-expiration (**B**) show pulmonary lobes with different colours and low attenuation areas as orange dots. At end-inspiration (**A**) the dots represent areas with attenuation values below -950 HU consistent with emphysema, whereas at end-expiration (**B**) the dots represent areas with attenuation values below -856 HU, consistent with air-trapping and emphysema.

## New horizons: analysis of exhaled gases, post-processing imaging techniques, and genetic analysis

The dramatically rising prevalence of respiratory diseases in older people requires that, as previously done for cognitive problems or heart failure, geriatricians become highly skilled in both traditional and innovative rapidly evolving diagnostic strategies for evaluating the airways.

The *analysis of exhaled gases* qualifies as a promising way of studying lung function. It can focus on selected exhaled gases, which can be determined through different analytical methods, such as high-performance liquid chromatography (HPLC) or mass spectrophotometry. Alternatively, a comprehensive non-qualitative picture of exhaled gases can be obtained by using the electronic nose, the so-called “e-nose” [46]. The ensuing breath pattern may be viewed as a sort of metabolic marker, reflecting non-respiratory diseases that impact the overall metabolism, e.g. liver failure and congestive heart failure. The available evidence on the e-nose shows an optimal diagnostic capacity for lung neoplasms, even at their initial stages [47]. Furthermore, the e-nose can help in classifying patients with COPD and can detect changes in breath pattern after nocturnal ventilation in patients with Obstructive Sleep Apnea [48]. The breath pattern measures have good reproducibility in both healthy and COPD subjects [49]. This dedicated tool collects the exhaled air and stores it, requiring the proband only to breath quietly through the mouthpiece, without the need of a nasal ring (European patent n. 2641537 (A1) ? 2013-09-25). This makes the procedure easy and comfortable, thus suitable even for the most frail and disabled older subjects. Noteworthy, the cost of air collection and examination may range is about 20 to 25$. This bulk of evidence makes the e-nose technology very promising for the study of older patients. Diagnostic applications of e-nose can be helpful in non-collaborating and disabled patients as well as in uncertain diagnoses of airway disease and in phenotypic characterization of certain chronic airway disease. Thus, it is likely that in the next future the e-nose may become a primary diagnostic tool in the older population.

As regards imaging, data obtained by a CT scan can be *post-processed* to generate high quality two-dimensional multiplanar reconstructions and 3D reconstructions that deliver a more anatomically meaningful display of the airways and improve communication among radiologists, clinicians, and patients [50, 51] (Fig. 4). 3D reconstructions include *CT bronchography*, an accurate roadmap of large airways, and virtual bronchoscopy, a simulation of the endoscopic navigation through the tracheobronchial tree (Fig. 4). In particular, *virtual bronchoscopy* represents a non-invasive alternative to conventional bronchoscopy in older adults and very sick patients and a non-invasive technique for long-term monitoring of tracheobronchial stents, with potential to replace conventional bronchoscopy in stent surveillance These techniques are not routinely performed in daily clinical practice yet, but nowadays they are commonly used for research purposes.

**Figure 6.**
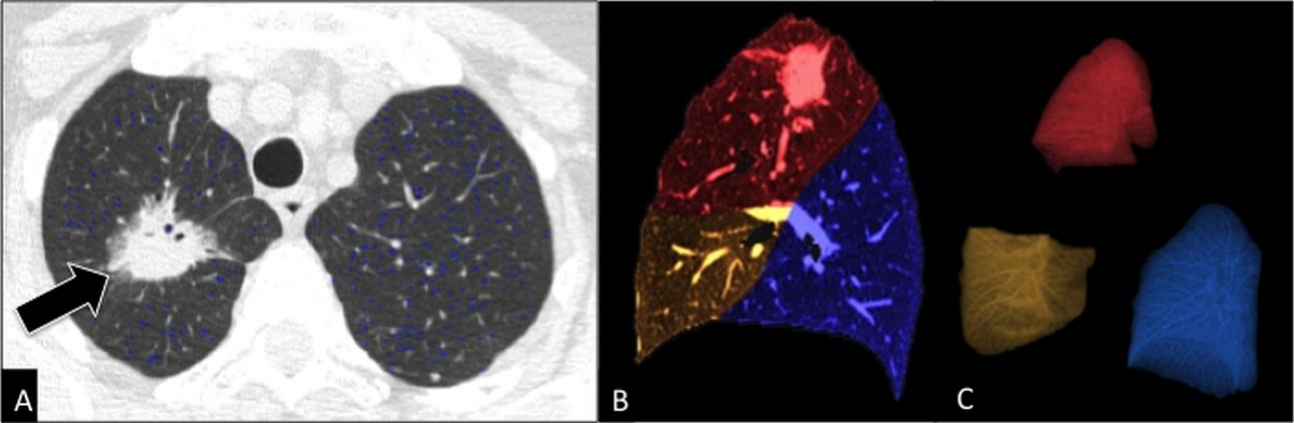
Post-processing imaging techniques: virtual lobectomy Axial CT image at lung window setting (**A**) shows a mass *(arrow)* in the apical segment of right upper lobe in a 73 years-old man, proved to be an invasive adenocarcinoma. Lung volume segmentation performed by using semi-automated software (Thoracic VCAR, GE Healthcare, Milwaukee, WI) allows to separate each lobe along the fissures (**B, C**) and to obtain the volume of emphysema (blue dots) within each lobe.

**Figure 7.**
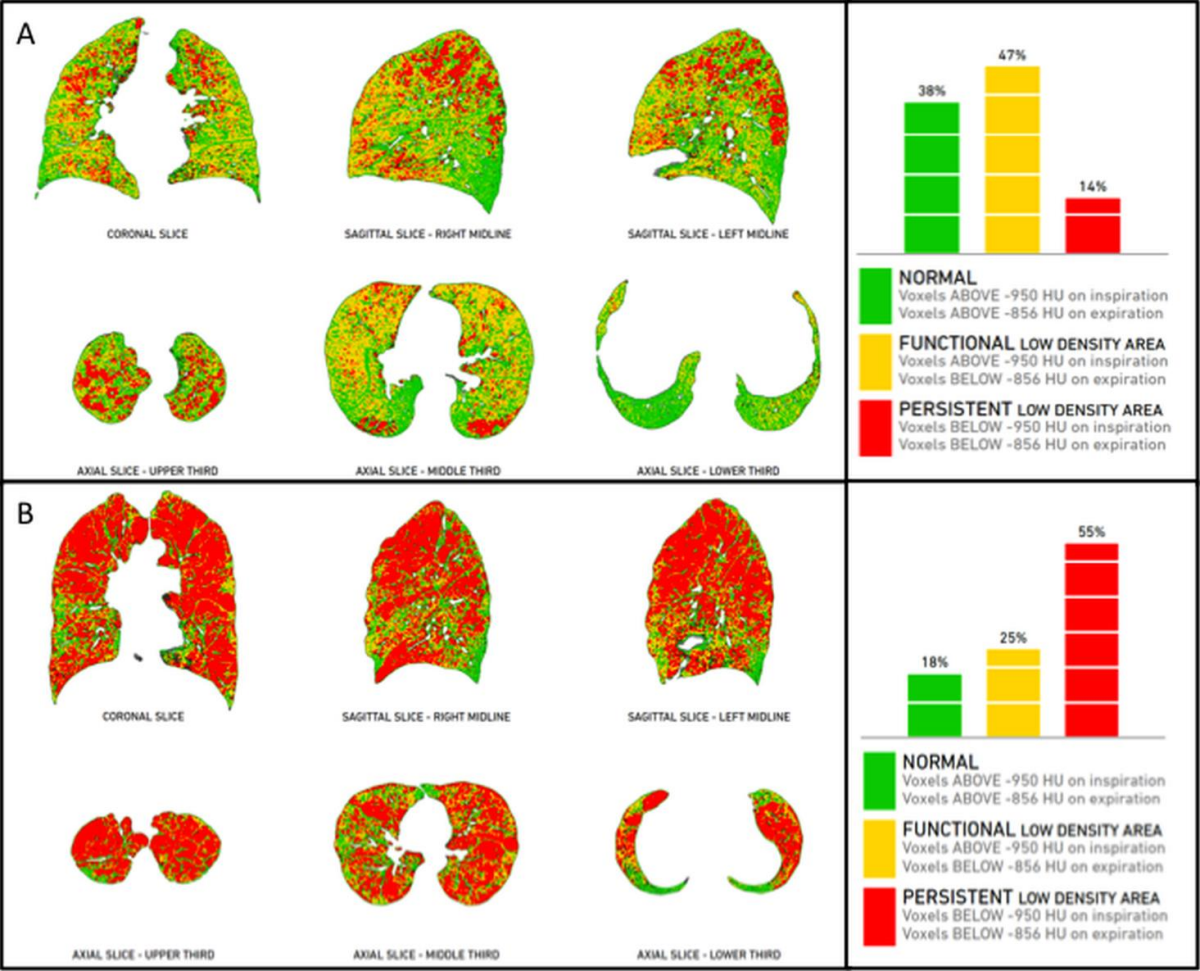
Post-processing imaging techniques: PRM in co-registration analysis Co-registration of inspiratory and expiratory CT scans (Imbio LLC, Minnesota, MN) provides a quantitative overview of the lungs (parametric response maps - PRM), with the relative volume of normal parenchyma (green), persistent airway disease (red), and functional airway disease (yellow). This innovative approach allows phenotypization of COPD without the need of an operator, as it is completely automated. Case “**A**” is a patient with predominant conductive airway disease (47% functional low-density area and 14% persistent low density area), whereas case “**B**” is a case with predominant emphysema (25% functional low density area and 55% persistent low density area).

Patients with COPD can take advantage of new diagnostic tools of automated detection and *quantification* of the extent of emphysema and expiratory air-trapping (Fig. 5), as well as changes in airway walls. The automated quantification of emphysema extent within each single lobe allows an accurate selection of preferred candidates for surgery or endoscopy. Those patients undergoing a surgical intervention for lobectomy, due to either asymmetric emphysema or lung tumors, may benefit from a pre-surgical evaluation of the remaining healthy as well as pathological lung volume by using a specific post-processing technique, called “*virtual lobectomy*” (Fig. 6). Furthermore, patients with COPD can nowadays receive a straightforward phenotypization of disease by using a *co-registration technique*, based on automated co-registration of inspiratory and expiratory CT scans [36, 52]. This technique provides radiologists and clinicians with a quantitative overview of the lungs in parametric response maps (PRM), with the relative volume of normal parenchyma, persistent airway disease, and functional small airway disease (PRM^FSA^) (see air-trapping in the section “Imaging findings between normal aging and clinically relevant airway disease”) (Fig. 7).

An integrated approach to both visual and quantitative assessment of CT scans is fundamental in evaluating patients with COPD, as described in the statement by the Fleischner Society on CT-definable subtypes of COPD [53]. The statement encourages such an approach to improve personalized care of patients, taking into account not only lung emphysema, but also coexisting findings: large airway disease, bronchiectases, interstitial lung disease, and pulmonary arterial enlargement.

Another innovative imaging analysis is the *fractal dimension analysis.* It has been performed on CT images to study the aged lung [54], revealing a significant decrease in lung complexity and lung density with age in never smokers. However, the exact histopathological basis for this finding in older individuals is unknown. Further studies on fractal dimensions are needed to investigate more specifically the aging airways.

*Ultrasounds analysis of diaphragm kinetics* has been evaluated as an additional imaging tool in assessing airway obstruction [55]. Indeed, the M-mode index of obstruction (ratio between forced expiratory diaphragmatic excursion in the first second and maximum expiratory diaphragmatic excursion) has been shown to represent a speed index of diaphragmatic relaxation, significantly correlated with FEV_1_/VC (p < 0.0001) [56]. An index value < 77 was identified as a possible cut-off for suspecting an obstructive airway disease (positive predictive value: 95.5%), thus representing a potentially helpful tool in screening obstructive airway disease in next future [56].

**Table 2:** Clinically relevant airway disease in older people: clinical relevance and role of imaging.

Airway disease	Clinical relevance in older people	Imaging
COPD	High prevalence in older people. Overdiagnosed if the normal threshold for adults is considered (FEV1/FVC <70%). Increasing role of imaging in phenotyping the disease. Challenge in distinguishing COPD from asthma cases and from cases with ACOS (Asthma-COPD Overlap Syndrome), common in older people [11].	Imaging may provide a structured morphometric analysis of both large and small airways, with the evaluation of parenchymal destruction, bronchial wall thickening as well as air-trapping. This approach allows distinguishing different phenotypes, such as predominant emphysema and predominant conductive airway disease. This distinction is the key for targeting patient therapy and selecting candidates for surgical or endoscopical therapy.
CPFE	Most often observed in 65 years-old men, cigarette smokers or ex-smokers of >40 pack-years [67]. PFTs show spurious preservation of lung volume together with depression of gas transfer.	CT shows centrilobular and/or paraseptal emphysema in upper zones and diffuse interstitial lung disease at the bases (subpleural reticular opacities, honeycomb, traction bronchiectasis, and more frequent ground-glass opacities than in idiopathic pulmonary fibrosis).
Bronchiolitis	Aspiration and infectious bronchiolitis are the most common forms in older people. Aspiration bronchiolitis is caused by recurrent aspiration of gastric contents or foreign bodies, common in older people. Infectious bronchiolitis is caused by immunodeficiency and ineffectiveness of mucociliary clearance, part of the physiological aging.	CXR shows diffuse, unilateral or bilateral, small (<5 mm) nodular lung opacities. CT shows centrilobular nodules and tree-in-bud opacities associated with adjacent areas of lobular consolidation. A patchy and asymmetric distribution is seen in infectious bronchiolitis, whereas dependent areas are more commonly affected in aspiration bronchiolitis.
Bronchiectases	Their diagnosis often clarifies otherwise unexplained respiratory symptoms. Older people are more prone to develop bronchiectases due to their immunodeficiency and weaken mucociliary clearance. Bronchiectases can cause recurrent tracheobronchial infections, hemoptysis, cough, systemic inflammation, respiratory failure. *Traction* bronchiectases are a relevant finding in interstitial lung disease.	CXR is often negative. CT is diagnostic, showing dilated and thickened bronchi without distal tapering. Cylindrical, varicose, or cystic appearance. Air-trapping, bronchial mucoid impaction, and pulmonary atelectasis can coexist. *Traction* bronchiectases are localized in areas of interstitial lung involvement, either within the reticular pattern or in the ground-glass pattern.
ECAC	Commonly underdiagnosed for its diagnostic challenge. ECAC includes tracheobronchomalacia and excessive dynamic airway collapse and it causes a worse respiratory quality of life. ECAC is more common in older subjects (mean age: 65±8.6) and in COPD patients [68].	In collaborating COPD patients with high clinical suspicion for excessive expiratory tracheal collapse a dynamic forced expiratory scan should be preferred to static end-expiratory scan [68]. On CT a reduction in cross-sectional tracheal area >50% is diagnostic in adults, with 70-80% cut-off being more specific [69]. However, no specific threshold of reduction in airway lumen has been suggested yet for older people.
Bronchial anthracofibrosis	Typical presentation in older women suffering from chronic cough, sputum, and dyspnea, and without a relevant history of pneumoconiosis or smoking [70].	CT scan shows multifocal stenoses of lobar and segmental bronchi in right upper lobe and right middle lobe, with peribronchial soft tissue thickening, calcified or non-calcified lymph nodes, and lobar or segmental atelectasis distal to the involved bronchi.
Broncholithiasis	Higher prevalence than younger adults because of high incidence of foreign body aspiration and calcified lymph nodes. Higher complication rate than younger adults. Complications like massive hemoptysis and obstructive pneumonia are very common in older people, causing significant morbidity and a difficult management.	CT scan shows calcified body (broncholith) within the lumen of the tracheobronchial tree along with post-obstructive findings, including bronchiectasis, consolidation and air-trapping.
Neoplasms	As age increases airway neoplasms more likely occur. However, benign airway neoplasms present with a higher incidence in older people. In two recent large series of primary *benign* airway tumors [71, 72] a higher occurrence in subjects older than 60 years was reported, but without a significant prevalence of any specific histotype. Endoscopical treatment can be curative in benign neoplasms.	CT scan shows intraluminal masses or airway wall thickening, with or without involvement of adjacent structures. CT is fundamental for staging and follow-up. *Benign* airway neoplasms are usually discovered incidentally on CT scans performed for other reasons.

(COPD= chronic obstructive pulmonary disease; CPFE= combined pulmonary fibrosis and emphysema; CT= Computed Tomography; ECAC= expiratory central airway collapse)

*Magnetic resonance imaging* (MRI) of the lung has been recently used in assessing COPD patients in comparison with PRM [57]. Ventilation defects at ^3^He MRI were quantitatively and spatially related to PRM gas-trapping in subjects with mild to moderate COPD, whereas they were related to both PRM gas-trapping and emphysema in subjects with severe COPD [57]. However, future studies should ascertain the spatial relationships between continuous pixel-wise data and PRM to provide a deeper insight of these relationships. Beyond COPD, lung MRI has also been used to evaluate the response to therapy in asthmatic patients using a turbo-inversion recovery-magnitude sequence. This sequence shows the extent of the segmental lung edema that corresponds to the severity of the regional allergic reaction, representing a promising biomarker for the non-invasive detection of the inflammatory response in asthmatic patients [58].

Another technique in combination between imaging and bronchoscopy is the *endobronchial optical coherence tomography* (EB-OCT), which can detect abnormalities of airway architecture in both large and medium-to-small-sized airways. Findings of airway remodeling have been found more pronounced in COPD patients, followed by smokers with normal FEV_1_ and never-smokers [59].

*Genomic studies* have recently been added in the spectrum of possible tools to better characterize COPD patients. In addition to the well-know deficiency of α1-antitrypsin as genetic cause of panlobular emphysema, several genome-wide associations studies (GWAS) have found specific loci on genes associated with COPD, including FAM13A, PID1, CHRNA3/5, IREB2, HHIP, AGER, SERPINA10, MOCS3, IFIT3, and DLC1 [60-64]. Noteworthy Cho MH et al [62] showed the associations between genes and imaging subtypes in 12,031 patients, opening a new horizon on the differential genetics of COPD phenotypes. However, GWAS are incapable of distinguishing causal from noncausal variants of COPD, thus suggesting the relevance of epigenetic investigations. By studying previously identified GWAS, DNA methylation profiling has identified FRMD4A, THSD4, and C10orf11 as the top differentially methylated sites [65]. Although several approaches (in vivo, in vitro, in silico) can be used for functional translation of genetic findings, this translation is still under investigation [66]. In next future identified genes and epigenetic variations might become effective drug targets or specific biomarkers for disease phenotypization.

## Conclusions

Nowadays a multidimensional assessment and interpretation of the aging airways can be used in our daily clinical practice by integrating clinical data together with morphologic and morphometric findings to disclose isomorphies (i.e. comparable trends of different dimensions) as to define the normal aging of the respiratory system and the disease status. Thus, a strict cooperative work between geriatricians, radiologists, and respiratory physiologists is highly desirable and many efforts should be done in this direction.

The multidimensional assessment in the context of a multidisciplinary approach lays the basis for a comprehensive geriatric assessment, a “technology of the geriatric care”, that optimizes the delivered care and inherent outcomes.

In next future, the implementation of the analysis of exhaled gases, post-processing imaging techniques, and genetic analysis of the older patient will hopefully close the gaps between normal aging and airway disease.
